# Periprosthetic Joint Infection Does Not Preclude Good Outcomes after a Revision Total Knee Arthroplasty: A 7-Year Follow-Up Study of 144 Retrospective Cases

**DOI:** 10.1155/2018/2582140

**Published:** 2018-08-12

**Authors:** Du Hyun Ro, Jong-Keun Kim, Sunghwan Kim, Hyuk-Soo Han, Myung Chul Lee

**Affiliations:** Department of Orthopaedic Surgery, Seoul National University College of Medicine, Seoul, Republic of Korea

## Abstract

**Background and Purpose:**

Debate exists on whether septic revision total knee arthroplasty (TKA) results in inferior clinical outcomes, and limited information is available regarding the factors associated with such outcomes. This study aimed to (1) compare clinical outcomes and characteristics of aseptic versus septic revision TKA and (2) identify the risk factors associated with inferior clinical outcomes.

**Methods:**

We retrospectively reviewed 144 revision TKAs (90 aseptic and 54 septic revisions) that were followed for a minimum of 3 years (mean = 7 years). Clinical outcome data, namely, Knee Society knee and function scores and the Hospital for Special Surgery knee score, were collected. We reviewed 13 pre- and intraoperative variables.

**Results:**

Postoperative clinical outcomes were inferior in septic revision surgeries (*p*<0.05). In regression analyses, however, septic revision was not an independent risk factor for poor clinical outcomes. The independent risk factors for poor outcome were identified where Anderson Orthopedic Research Institute grade 3 femoral and tibial bone defects, more than three surgeries, and treatment for persistent infection were associated with inferior clinical outcomes (all* p*<0.05). Standard two-stage septic revision without grade 3 bone defects or additional surgeries showed comparable outcomes to aseptic revision.

**Interpretation:**

Clinical outcomes of septic revision were inferior to those of aseptic revision. However, poor outcomes were mainly associated with large bone defects and an increased number of surgeries. The outcomes of aseptic and septic revision surgery were similar when patients with larger bone defects and more than three surgeries were excluded.

## 1. Introduction

More than 650,000 total knee arthroplasties (TKAs) are performed annually in the United States [1]. As the geriatric population increases, the number of TKAs is expected to increase; subsequently, the demand for revision surgery will also increase [1]. Revision surgery is a complex, demanding procedure and, importantly, clinical outcomes are less satisfying than those of primary TKA [2, 3].

It is generally accepted that the etiology of revision surgery influences the outcome. Of the major etiologies for revision surgery, septic revision is associated with the worst outcome [2–8]. Before the introduction of two-stage revision surgeries, eradication of infection was less common; and consequently clinical outcomes were rather poor [9].

As treatment strategies for septic revision have improved, identification of organisms, eradication rates, and clinical outcomes have also improved impressively [10–14]. Some authors have reported that septic and aseptic revision groups have had similar outcomes regarding pain, functional scores, survival, and mental health status [10–12]. Patil et al. even reported a higher clinical score with septic versus aseptic revisions [11]. Recent literature has suggested that when a standard protocol and team-based approach are used, periprosthetic infection does not preclude a good outcome after revision TKA [10–12]. Hence, there is now debate regarding whether septic revision is associated with poor clinical outcomes.

Such debate regarding the clinical outcomes of septic versus aseptic surgery suggested to us that focusing on the “cause of revision” may mean that something more important is missed. Of note, revision surgeries comprise diverse clinical situations. Unlike primary TKA, revision surgeries are associated with various degrees of bone defect, different implant configurations, the use of a more extensive surgical approach, and the need for repeat surgeries [7, 15, 16]. Hence, we hypothesized that the cause of the revision, especially infection, could be a confounding factor and that other unknown factors associated with infection could be more directly related to clinical outcomes. However, limited information is currently available regarding the factors associated with inferior clinical outcomes in revision TKAs with mid-to long-term follow-up. Information regarding this subject would help to improve the clinical outcome of revision TKA. Therefore, this study aimed to (1) compare clinical outcomes of aseptic versus septic revision TKA and (2) identify risk factors associated with inferior clinical outcomes.

## 2. Materials and Methods

### 2.1. Study Subjects

**Figure 1:**
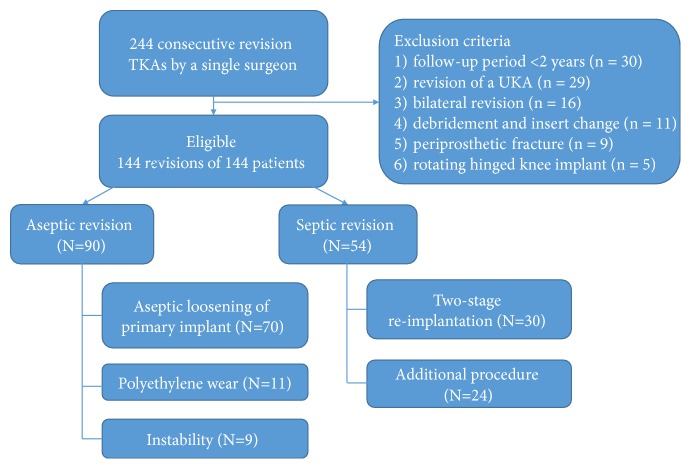
Flow chart of the study subjects.

This retrospective study was approved by our local Institutional Review Board (Protocol No: 1307-114-506). We reviewed a single institution database of 244 consecutive revision TKAs performed by a single surgeon from 1995 to 2015. Based on the following criteria, 100 revisions were excluded: (1) follow-up period less than 2 years or loss (n = 30); (2) revision of a unicompartmental knee arthroplasty (n = 29); (3) bilateral revision (n = 16); (4) acute hematogenous infection that was successfully treated with debridement and insert change (n = 11); (5) periprosthetic fracture that required revision TKA (n = 9); and (6) rotating hinged knee implant (n = 5). This left 144 revisions of 144 patients (Figure 1). The study group included 20 males and 124 females with an average age of 68.4 years (range: 50–83 ± 7.2 years). The aseptic revision group included 70 cases with aseptic loosening of the primary implant, 11 cases with polyethylene wear, and 9 cases with instability. Both component revisions (Femur and Tibial component) were performed for all aseptic revisions. The septic revision group included 30 cases with chronic infection that were treated with two-stage reimplantation and 24 cases that underwent an additional arthrotomy and debridement for persistent infection, before or after two-stage reimplantation.

The average length of follow-up after revision was 84 ± 28.7 months (range: 40–168 months), and the average interval between the primary and revision surgeries was 99 ± 58.7 months.

**Figure 2:**
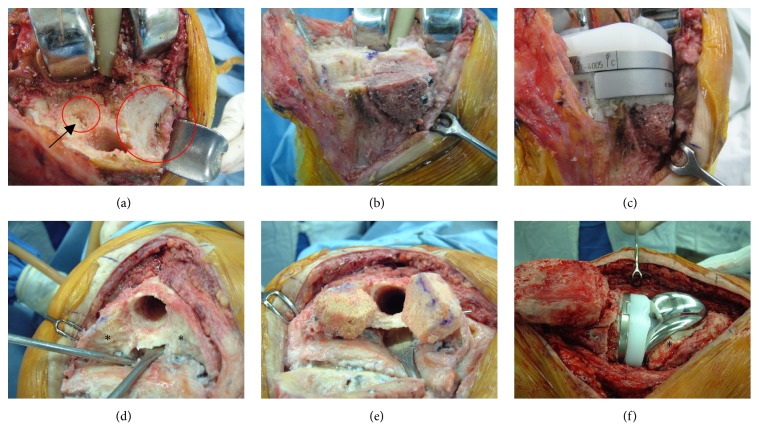
Bone defect filling process. (a) Intraoperative photograph showing an unconstrained, large bone defect with destruction of the metaphyseal bone (grade 3 bone defect) on the medial side of the tibia (asterisk) and a small, constrained bone defect (arrow) on the lateral side of the tibia. (b) Initially the large bone defect was treated with allografts using screw fixation in the metaphysis. (c) Then the remaining unconstrained bone defect was filled with a metal block and the defect on the lateral side was filled with cement. (d) Intraoperative photograph showing unconstrained large bone defect (asterisks) with destruction of major metaphyseal bone in femur (grade 3 bone defect). (e) Allograft was also used to fill the large bone defect. (f) Implant was inserted to the augmented area and the allograft was tightly compacted between femur component and remaining bone (asterisk).

During the study period, revision surgery was performed with either a varus-valgus constrained implant (LCCK®, NexGen®, Zimmer, Warsaw, IN, USA) or a posterior stabilized implant (LPS®, NexGen®, Zimmer, Warsaw, IN, USA), depending on the stability [16]. A fluted titanium extension stem, titanium block, and/or strut allograft were used, depending on the bone defect. Contained bone defects <5 mm thick were filled with bone cement. Uncontained bone defects ≤10 mm thick were treated with block augment and uncontained bone defects >10 mm thick were treated with strut allografts using screw fixation (Figure 2). Stem extensions were fixed using the hybrid fixation technique for the entire implant. Intraoperative observations were systemically collected using a predesigned database. Bone defects were classified according to the Anderson Orthopedic Research Institute (AORI) bone defect protocol [17]. Two independent investigators prospectively collected all the clinical information using the predesigned computer database (SMA and EMS). Basic demographic data and clinical outcomes, including the Knee Society Knee score (KSKS) and Knee Society function score (KSFS), and the Hospital for Special Surgery knee score (HSS) were recorded. Postrevision outcomes were collected annually and the most recent follow-up data were used. ROM was measured from maximum extension to maximum flexion using a standard clinical goniometer with the patient in the supine position.

### 2.2. Protocol of Septic Revision Surgery

For patients with a chronic periprosthetic infection, a two-stage reimplantation was performed, which included removal of components, extensive debridement, and placement of an antibiotic-impregnated articulating cement spacer, followed by 6 to 8 weeks of intravenous antibiotics according to the microorganism. After a 4-week antibiotic free interval, we performed laboratory tests and joint aspirations to determine whether the infection has been eradicated. If so, reimplantation was performed. Debridement and replacement of the antibiotic-impregnated cement spacer were performed instead of reimplantation if there were signs and symptoms of persistent infection. The criteria for a persistent infection included ongoing discharge and erythema, higher than 36.5°C of the body temperature, higher than 0.5 mg/dl of the C-reactive protein level, or more than five polymorphonuclear neutrophils observed on any high-power field (HPF) in 10 frozen section specimens harvested intraoperatively from the synovium or necrotic tissue debris.

### 2.3. Statistical Analysis

Clinical outcomes and characteristics of the septic revisions were compared with those of the aseptic revisions using Student's t-test for continuous, normally distributed data and Pearson's chi-square test for nominal, categorical data. Within both groups, the prerevision and postrevision data were compared using the paired t-test for the normally distributed data. Normality of data was assessed using the Kolmogorov-Smirnov test. For all analyses, the level of significance was set at a* p* value of <0.05.

To identify the factors associated with inferior clinical outcomes in revision TKAs, linear regression analyses were used. Thirteen variables were assessed, including age, sex, body mass index (BMI), the primary diagnosis (0, osteoarthritis; 1, rheumatoid arthritis), the cause (0, aseptic loosening; 1, septic loosening), prerevision ROM, implant type (0, PS implant; 1, LCCK), the surgical approach (0, standard parapatellar approach; 1, quad-snip; 2, V-Y quadriceps plasty; and 3, tibial tubercle osteotomy), the femur bone defect (AORI type 1, 2, or 3), the tibia bone defect (AORI type 1, 2, or 3), complications (0, no complication; 1, a complication present), insert thickness, and number of operations (arthrotomy operation). Factors with a p value < 0.20 on univariate analysis were assessed subsequently through multivariate analysis using the stepwise method. Statistical analyses were performed using the SPSS® for Windows® statistical software package (ver. 19.0.1; SPSS Inc., Chicago, IL, USA).

## 3. Results

**Figure 3:**
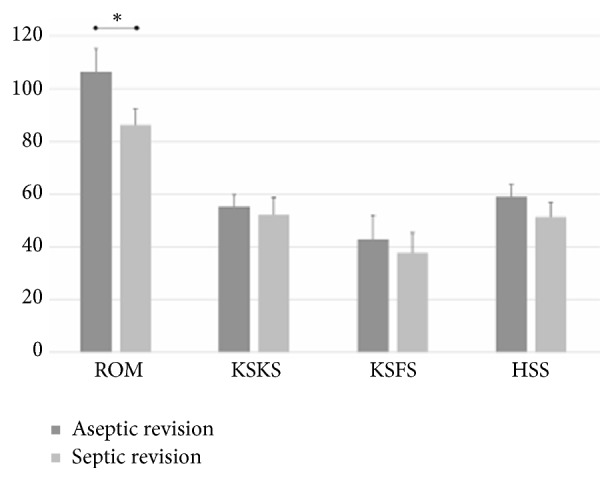
Characteristics and clinical scores of each revision group. Preoperative ROM was significantly greater in the aseptic revision group. Values are means and standard deviations, and the asterisk denotes statistical significance. ROM, range of motion; KSKS, Knee Society knee score; HSS, Hospital for Special Surgery knee score.

**Figure 4:**
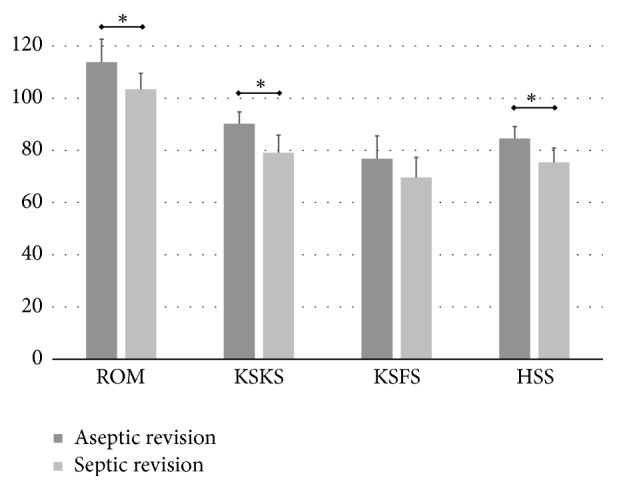
Postoperative outcomes of each revision group. Postoperative ROM, KSKS, and HSS were significantly lower in the septic revision group. Values are means and standard deviations, and asterisks denote statistical significance. ROM, range of motion; KSKS, Knee Society knee score; HSS, Hospital for Special Surgery knee score.

The preoperative clinical outcomes were similar between the aseptic versus septic revision groups (Figure 3). However, the preoperative ROM was higher in the aseptic revision group (p<0.001). In both groups, all clinical outcomes (KSKS, KSFS, and HSS scores) improved after revision (*p<*0.001) as did the ROM. However, the final scores were less satisfying in the septic revision group. The postrevision ROM, KSKS score, and HSS score were significantly lower (p=0.030, <0.001, and 0.003, respectively). Only the KSFS score was similar between the two groups (p=0.105, Figure 4).

Regarding the pre- and intraoperative factors, constrained implant was more frequently used in the septic revision group (p<0.001) and a more extensive approach was chosen (*p*=0.008) (Table 1). Repeated surgery was required in the septic revision group (*p<0.001*) and complications were more frequent (p=0.011). Also, the femoral bone defects tended to be more extensive (*p*<0.001).

Univariate and subsequent multivariate linear regression analyses were performed to identify factors that were associated with clinical outcomes and ROM (Tables 2 and 3). Regression analysis revealed that the postrevision ROM increased with age (*p=*0.001) and greater prerevision ROM (*p<*0.001) and decreased with tibia bone defects (both grades 2 and 3,* p<*0.001 and* p*<0.002, respectively) and three or four surgeries (*p<*0.001). We performed the same analysis for the remaining clinical outcomes and showed that femoral bone defects (grade 3), tibial bone defects (grade 3), and the three or four surgeries were strongly associated with inferior clinical outcomes in revision TKA. Specifically, the KSKS, KSFS, and HSS scores were related to grade 3 femoral bone defects, ROM and HSS to grade 3 tibial bone defects, and ROM, KSKS, and HSS scores to having three or four surgeries. However, the cause of revision was not associated with the clinical outcomes.

**Figure 5:**
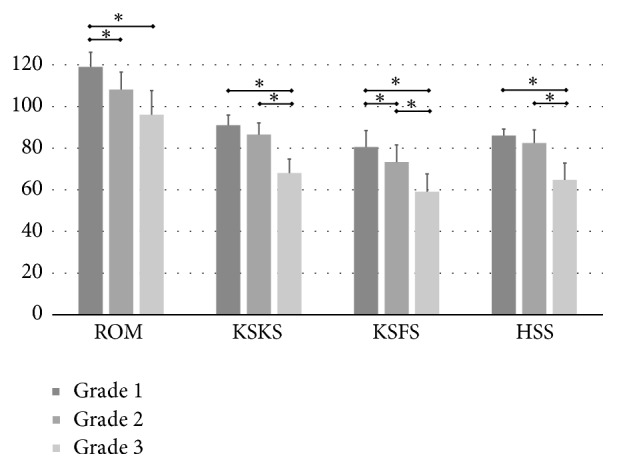
Clinical outcomes according to the severity of the femoral bone defect. Grade 3 bone defects showed inferior outcomes compared to grade 1 and 2 defects. Values are means and standard deviations, and asterisks denote statistical significance. ROM, range of motion; KSKS, Knee Society knee score; HSS, Hospital for Special Surgery knee score.

**Figure 6:**
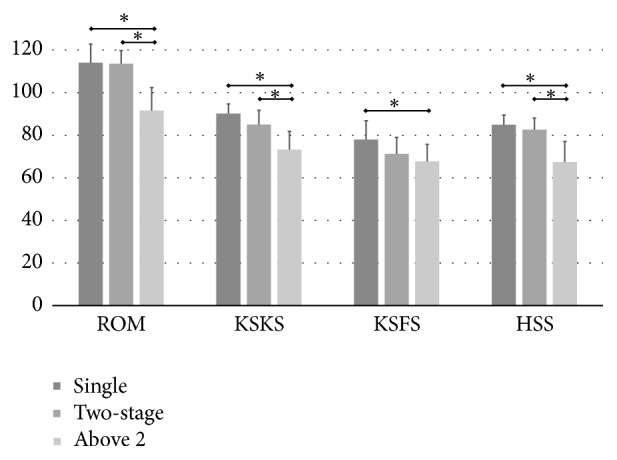
Clinical outcomes according to the number of surgeries. Outcomes were inferior with more than two surgeries compared with single- or two-stage surgery. Values are means and standard deviations, and asterisks indicate statistical significance. ROM, range of motion; KSKS, Knee Society knee score; HSS, Hospital for Special Surgery knee score.

Postrevision clinical outcomes were compared according to bone defect and number of surgeries. As the degree of defective femoral bone increased, the ROM and outcome scores gradually decreased (Figure 5). The clinical outcomes of patients with a grade 3 bone defect were especially poor for every outcome score (*p<0.05*). Regarding the number of surgeries, three or four surgeries had significantly inferior outcomes (*p<0.05*). However, patients that had only a two-stage revision in the absence of large bone defects did similar to aseptic revisions (Figure 6).

## 4. Discussion

The most important finding of our study was that inferior clinical outcomes in revision TKA surgery were related to large bone defects (grade 3) and greater numbers of surgeries (more than three) and not with the type of revision (septic vs. aseptic revision). Septic revision was not directly related to clinical outcomes per se; instead it was indirectly related with an increased number of surgeries and larger bone defects, which are characteristics of persistent infection after failure to control the initial infection. Our data showed that the outcomes of aseptic and septic revision surgery were similar if patients with larger bone defects and more than three surgeries were excluded.

There is debate regarding whether septic revision results in inferior clinical outcomes [2–8, 10–12]. Barrack et al. reported that septic revision was associated with significantly lower functional scores [4]. However, patients undergoing second or third revisions were included. Van Kempen et al. reported a similar result, but they did not report on the bone defects encountered or the number of revisions [8]. Patil et al. reported higher clinical scores after septic revisions than after aseptic revisions [11]. However, they included polyethylene exchange and chronic osteomyelitis patients in their analyses. We believe that these inconsistent results originate from the complexity of revision surgery. Most previous reports simply compared septic versus aseptic revision surgery. However, septic revision encompassed various clinical situations, including large bone defects and even failed infection control. It is not “inappropriate” to state that septic revisions have poorer outcomes. That is a fact. However, it is not the infection that is an independent risk factor but rather the size of the bone defect and the number of surgeries.

Limited information is available regarding the factors affecting clinical outcomes in revision surgery. It has been reported that aggressive microorganisms, chronic lymphedema, repeated surgery, and comorbidities increase the failure risk of revision surgery and also result in poor clinical outcomes [18–20]. Although previous studies have focused mainly on the success rate of revision surgery, we believe their findings are in line with our research.

Extra surgical procedures over a two-stage surgery (3rd or 4th surgery) resulted in inferior clinical outcomes. These patients had a decreased ROM as well as poorer functional and pain scores. Repeated tissue injury that results in persistent inflammation with tissue degeneration and emotional depression due to prolonged hospitalization may lead to inferior clinical outcomes for these patients [21–23]. In fact, we often see patients who are depressed and disappointed that the infection was not controlled even after debridement or a two-stage surgery. It is notable that extra surgical procedures (more than two-stage) most commonly occurred due to failed infection control. Sherrell et al. reported that failure of irrigation and debridement leads to subsequent failure of two-stage reimplantation and ultimately requires another operation for persistent infection [24]. Our findings suggest that failure to control infection in a two-stage surgery or an inappropriate treatment decision for periprosthetic infection may result in poor clinical outcomes. We would expect satisfactory clinical outcomes for septic revision with a standard treatment protocol.

Several studies have reported poor outcomes for revision TKA of larger bone defects. Franke et al. reported that 20% of patients experienced a poor outcome in their 5-year follow-up, and Clatworthy et al. reported a 72% 10-year success rate, meaning that one of four patients required re-revision surgery [25, 26]. In our case, there were 7 failures among 18 grade 3 femoral bone defects. The most common reason was loosening of the implant, of which there were three cases that eventually required re-revision. The second most common reason was infection. Two patients required arthrodesis. The third most common reason was instability, as two patients required a knee brace but they declined a further procedure. Reconstruction techniques other than an allograft should be considered to solve these problems. Although it is currently unclear why femoral bone defects were more related to a poor outcome than were tibial bone defects, efforts should be made to reduce bone defects, especially of the femur, during revision TKA. In our experience, a motorized burr is better than a curette and osteotome for preserving healthy bone during debridement.

Readers should be aware of several limitations of the current study. First, due to the retrospective nature of the study and the scarcity of revision cases, we could not effectively control the baseline demographics. Thus, gender and BMI in this study differed between the aseptic and septic groups, introducing the possibility of selection bias. Although a matched study would be more desirable, it is actually impossible to match perfectly or stratify subjects while maintaining statistical power in a revision study. Thus, we used multiple regression analysis to correct for confounding bias of independent variables affecting the clinical outcomes. Despite the limitations, the statistical power of our regression model was sufficient to validate our outcome. Second, a considerable amount of variance in our multivariate model remained unexplained. This indicates that other unknown factors, such as combined spine pathology, general health status, quadriceps muscle strength, presence of microorganisms, and mental health, may have been related to the clinical outcomes [22, 23, 27, 28]. However, the value of such information is limited as these variables cannot be modified during the surgical procedure. We believe that our evaluation of 13 variables included most of the intraoperative and surgically correctable factors and provided information that was relevant to improving the clinical outcome. Third, the female predominance of the study population should be noted. The proportion of females was 83.8%, which was substantially higher than that reported by other studies of outcomes of revision surgery [2–8, 10–12, 29, 30]. Although there is no clear explanation for the female predominance in knee osteoarthritis, it has been consistently reported in several epidemiologic studies [31, 32]. This predominance is even greater in Koreans; consequently, the incidence of TKA is 7-8-fold higher in females than in males [33]. This could explain the predominance of females in this study and indicates that the possible selection bias was negligible.

## 5. Conclusions

Clinical outcomes of septic revision were inferior compared to those of aseptic revision. However, poor outcomes mainly resulted from large bone defects and a high number of surgeries. The outcomes of aseptic and septic revision surgery were similar when patients with larger bone defects and more than three surgeries were excluded from the analyses.

## Figures and Tables

**Table 1:** Characteristics of the study groups.

	Aseptic revision	Septic revision	P-value
	(n = 90)	(n = 54)	
	Mean ± SD	Mean ± SD	
Age (years)	69.1 (50–83)	67.2 (50–80)	0.095
Female gender	85 (94.4%)	39 (72.2%)	<0.001
Body mass index (kg/m^2^)	28.0 ± 4.4	25.9 ± 3.6	0.007
Average polyethylene thickness (mm)	16.6 ± 3.29	16.2 ± 3.3	0.621
Varus-valgus constrained implant	61 (67.8%)	50 (92.6%)	<0.001
Surgical approach			
Standard parapatellar approach	64 (71.1%)	26 (48.1%)	0.008
Extensive approach	26 (28.9%)	28 (51.9%)	
Bone defect			
Grade 1 / 2 / 3 femoral bone defect	30 / 55 / 5	4 / 37 / 13	<0.001
Grade 1 / 2 / 3 tibial bone defect	32 / 53 / 5	20 / 27 / 7	0.256
Average number of surgeries	1.01	2.7*∗*	<0.001
Wound complications	1 case	6 cases	0.011
Average time interval between primary and revision surgery (months)	127 ± 35	53 ± 28	<0.001

Values are means ± standard deviations or percentages.

*∗*Two-stage revision, 30 cases; three- or four-stage revision, 24 cases.

**Table 2:** Results of univariate regression analysis including 13 pre- and intraoperative variables and four clinical outcomes.

	ROM		KSKS		KSFS		HSS	
Variable	*β* ± SE*∗*	P-value	*β* ± SE*∗*	P-value	*β* ± SE*∗*	P-value	*β* ± SE*∗*	P-value
(1) Age	0.8±0.2	<0.001	0.1±0.2	0.339	0±0.2	0.891	0.2±0.2	0.233
(2) Female gender	1.5±4.6	0.742	3.9±3.2	0.227	0.6±4.3	0.885	0.7±3.3	0.842
(3) Body mass index	0.5±0.4	0.259	0±0.3	0.996	0±0.4	0.919	–0.1±0.3	0.837
(4) Primary diagnosis Osteoarthritis (comparator)								
Rheumatoid arthritis	–7.2±6.6	0.279	–1.9±4.8	0.697	–7.5±6.5	0.249	–7.4±4.9	0.132
Other	–3.6±8	0.659	3±5.5	0.590	2.2±7.5	0.771	4.5±5.6	0.425
(5) Cause	–5.3±1.6	0.001	–4.8±1.1	<0.001	–3.7±1.5	0.017	–4.7±1.1	<0.001
(6) Preoperative ROM	0.3±0.1	<0.001	0.1±0	0.014	0.1±0.1	0.028	0.1±0	0.016
(7) Implant	–2.8±3.8	0.460	–3.8±2.6	0.145	–8.4±3.5	0.016	–5.6±2.6	0.034
(8) Standard paramedian approach (comparator)								
Quadriceps snip	–7±3.6	0.056	–6.9±2.5	0.006	–10.5±3.3	0.002	–5.2±2.6	0.046
VY quadriceps plasty	–1±5.8	0.866	7.3±3.9	0.066	11.2±5.3	0.037	8±4	0.049
Tibial tubercle osteotomy	–8.4±6.6	0.207	–5.7±4.5	0.208	1.2±6.2	0.85	–7.7±4.9	0.118
(9) Grade 1 femur bone defect (comparator)								
Grade 2	–5.1±3.3	0.125	0.9±2.3	0.696	–2.6±3.1	0.397	0.1±2.3	0.955
Grade 3	–21.5±4.8	<0.001	–21.6±3.1	<0.001	–18.5±4.5	<0.001	–18.7±3.2	<0.001
(10) Grade 1 tibial bone defect (comparator)								
Grade 2	–10±3.1	0.002	–4.3±2.2	0.051	–8.6±2.9	0.018	–5.2±2.2	0.022
Grade 3	–20.8±6.4	0.001	–13.7±4.4	0.002	–13.7±6.1	0.026	–11.8±4.8	0.016
(11) Insert thickness	0.4±0.6	0.499	0.1±0.4	0.780	0.3±0.5	0.562	0±0.4	0.978
(12) Wound complications	–16.6±7.9	0.038	–8.7±5.5	0.115	–4.4±7.5	0.555	–18.8±5.4	0.001
(13) Single surgery (comparator)								
Two-stage	5.2±3.9	0.19	–2.1±2.7	0.448	–5.2±3.7	0.164	1.5±2.8	0.584
Three- or four-stage	–22.3±3.8	<0.001	–15.5±2.7	<0.001	–7.9±3.9	0.025	–16.9±2.6	<0.001

*∗*Values are standardized regression coefficients (*β*) ± standard errors (SE). ROM, range of motion; KSKS, Knee Society knee score; HSS, Hospital for Special Surgery knee score; KSFS, Knee Society function score

**Table 3:** Results of multivariate regression analysis: relationships between selected variables and four clinical outcomes*∗*.

Multivariate analysis	ROM		KSKS		KSFS		HSS	
Variable	*β* ± SE^†^	P-value	*β* ± SE^†^	P-value	*β* ± SE^†^	P-value	*β* ± SE^†^	P-value
Age	0.6±0.2	0.001						
Cause of revision								
Preoperative ROM	0.2±0.1	<0.001						
Grade 1 femur bone defect (comparator)								
Grade 2			–5.8±2.0	0.005	–10.2±3.2	0.002		
Grade 3			–21.6±3.4	<0.001	–25.6±4.9	<0.001	–12.9±3.1	<0.001
Grade 1 tibial bone defect (comparator)								
Grade 2	–12.9±2.5	<0.001						
Grade 3	–16.7±5.3	0.002					–5.5±1.9	0.004
Single surgery (comparator)								
Two-stage								
Three- or four-stage	–16.1±3.3	<0.001	–10.2±2.5	<0.001			–13. 3±2.5	<0.001
R^2^_adj_^‡^	0.45		0.36		0.16		0.34	

*∗*Variables with p<0.20 in the univariate analysis were included in the multivariate analysis (stepwise method). Nonsignificant factors were excluded from the table. ROM, range of motion; KSKS, Knee Society knee score; HSS, Hospital for Special Surgery knee score.

^†^Values are *β*  ±  SE.

^‡^R^2^_adj_, percent variance explained by each variable.
